# Immune responses in healthy adults elicited by a bivalent norovirus vaccine candidate composed of GI.4 and GII.4 VLPs without adjuvant

**DOI:** 10.3389/fimmu.2023.1188431

**Published:** 2023-06-26

**Authors:** Gwenn Waerlop, Yorick Janssens, Bart Jacobs, Franziska Jarczowski, André Diessner, Geert Leroux-Roels, Victor Klimyuk, Isabel Leroux-Roels, Frank Thieme

**Affiliations:** ^1^ Center for Vaccinology (CEVAC), Ghent University and University Hospital, Ghent, Belgium; ^2^ Icon Genetics GmbH, a Denka Company, Halle, Germany

**Keywords:** norovirus, virus-like particle, plant-produced, vaccine, clinical trial, immunogenicity

## Abstract

The development of an efficacious vaccine against norovirus is of paramount importance given its potential to reduce the global burden of norovirus-associated morbidity and mortality. Here, we report a detailed immunological analysis of a phase I, double-blind, placebo-controlled clinical trial performed on 60 healthy adults, ages 18 to 40. Total serum immunoglobulin and serum IgA against vaccine strains and cross-reactive serum IgG against non-vaccine strains were measured by enzyme immunoassays, whereas cell-mediated immune responses were quantified using intracellular cytokine staining by flow cytometry. A significant increase in humoral and cellular responses, e.g., IgA and CD4^+^ polypositive T cells, was triggered by the GI.4 Chiba 407 (1987) and GII.4 Aomori 2 (2006) VLP-based norovirus vaccine candidate rNV-2v, which is formulated without adjuvant. No booster effect was observed after the second administration in the pre-exposed adult study population. Furthermore, a cross-reactive immune response was elicited, as shown by IgG titers against GI.3 (2002), GII.2 OC08154 (2008), GII.4 (1999), GII.4 Sydney (2012), GII.4 Washington (2018), GII.6 Maryland (2018), and GII.17 Kawasaki 308 (2015). Due to viral infection *via* mucosal gut tissue and the high variety of potentially relevant norovirus strains, a focus should be on IgA and cross-protective humoral and cell-mediated responses in the development of a broadly protective, multi-valent norovirus vaccine.

**Clinical trial registration:**

https://clinicaltrials.gov, identifier NCT05508178. EudraCT number: 2019-003226-25.

## 1 Introduction

Noroviruses are the leading cause of sporadic and epidemic acute non-bacterial gastroenteritis and foodborne diarrheal diseases in humans worldwide, resulting in an estimated 669 million illnesses and 219,000 deaths across all age groups each year (1, 2). This is associated with an estimated total cost of 4.2 billion US dollars in direct health system costs and 60.3 billion US dollars in societal costs (3). Severe outbreaks typically occur in close-quartered environments such as hospitals, military barracks, schools, camps, and ships (4–7). Norovirus particles exhibit high environmental stability on exposed surfaces and are mainly transmitted through the fecal/oral route, with infection typically characterized by severe vomiting, diarrhea, and abdominal cramping for 28–60 h within 10–51 h of exposure (8, 9).

Noroviruses are a group of non-enveloped, positive-sense, single-stranded RNA viruses. Their genome consists of three open reading frames (ORFs), with ORF2 encoding the major capsid protein VP1, which is the determining factor for the virus’s antigenicity and can be used to classify noroviruses into 10 genogroups (GI to GX) and 49 confirmed genotypes (10, 11). Of these, the GI and GII genogroups have been found to be responsible for the majority of human diseases, and GII.4 viruses have caused the majority of norovirus outbreaks in the past two decades (10).

No approved vaccines against norovirus are currently available, making the development of an efficacious vaccine to prevent severe norovirus gastroenteritis a high priority considering the burden of disease caused by norovirus. Several candidate vaccines are currently under development, with projections indicating that they could have both clinical and economic benefits, including reduced healthcare costs and decreased absenteeism (12). However, the development of such a vaccine is hampered by factors such as an incomplete understanding of protective immunity to norovirus (lack of defined correlates of protection), a lack of a permissive cell culture system and suitable animal models, and the complex landscape of norovirus strains infectious to humans (13). The most advanced candidate vaccines are based either on virus-like particles (VLPs) composed of norovirus capsid protein VP1 formulated with alum adjuvant for intramuscular delivery or on recombinant adenovirus expressing VP1, adjuvanted with dsRNA, and formulated as tablets for oral delivery (13–15). Clinical trials using VLP-based vaccines in adult volunteers (16–19), older adults (20), and children (21) have demonstrated their safety and immunogenicity.

This publication provides a more in-depth evaluation of the immune responses observed in our clinical trial of the first plant-produced, prophylactic vaccine candidate manufactured in Europe (22). The goals of this trial were to evaluate the safety and immunogenicity of a non-adjuvanted, bivalent (GI.4 Chiba 407 [1987] + GII.4 Aomori 2 [2006]) vaccine consisting of VLP at two dose levels (50 µg or 150 µg each) manufactured using the magnICON^®^ plant-based, transient expression system (23, 24). Safety and initial evaluation of immune responses for the 60 healthy adult participants of the trial were already published in Leroux-Roels et al. (22). Here a more in-depth analysis of the immune responses, including vaccine-strain-specific total immunoglobulin, IgA, and further evaluation of cross-reactive IgG against non-vaccine strains, is provided. Additionally, quantification of cell-mediated responses using intracellular cytokine staining (ICS) by flow cytometry is presented and discussed.

## 2 Materials and methods

### 2.1 Study vaccine, study design, and participants

The recombinant norovirus vaccine candidate, rNV-2v, consists of VLPs self-assembled from the major capsid proteins (VP1) of the GI.4 Chiba 407 (1987) and GII.4 Aomori 2 (2006) strains, respectively. Manufactured by Icon Genetics GmbH using its magnICON^®^ technology, the candidate vaccine was tested in a randomized, placebo-controlled, double-blind, dose-escalating Phase I study involving 60 healthy men and women aged 18–40, randomly assigned to two cohorts receiving either low- or high-dose vaccine (50/50 or 150/150 µg VLP GI.4 and GII.4, respectively). More detailed information is provided by Leroux-Roels et al. (22).

A vaccine or placebo was administered in the deltoid muscle with a 28-day interval between the two doses. Humoral immune responses were measured using serum samples prepared from venous blood ( ± 12 ml) drawn on day 1 (before the first administration), day 8, day 29 (before the second administration), and subsequently on days 57, 183, and 365. Serum was prepared and kept frozen at -20°C until analysis by ELISA. Cellular immune responses were measured using peripheral blood mononuclear cells (PBMC) isolated from heparinized venous blood samples ( ± 50 ml) collected on days 1, 29, 57, and 365. PBMC were isolated by isopycnic density centrifugation (Ficoll–Hypaque) and stored at -196°C in liquid nitrogen until analyzed.

### 2.2 Serum antibodies against vaccine VLPs

Plant-produced VLPs of GI.4 Chiba 407 (1987) and GII.4 Aomori 2 (2006) (Icon Genetics GmbH) were coated onto separate 96-polystyrene plates (Nunc MaxiSorp) by adding 100 µl per well of a coating solution (GI.4: 4 µg/ml for IgA and 2 µg/ml for total Ig; GII.4: 4 µg/ml for both). After washing, the plates were blocked for 2 h using skim milk, followed by sample incubation for 2 h. After this, plates were incubated for 1 h with horseradish peroxidase-conjugated antibodies against either IgA or total Ig (HRP Goat anti-human IgA, BioLegend; Goat anti-human Ig : HRP, BIO-RAD), followed by 15 min (total Ig) or 30 min (IgA) incubation with the chromogen substrate (3,3′,5,5′-Tetramethylbenzidine, TMB). The coloring reaction was stopped by adding 1N H_2_SO_4_. Between every step, the plates were washed with PBS (phosphate-buffered saline). Plates were read at 450 nm using a Versamax microplate reader (Molecular Devices). Optical density (OD) values were processed with data reduction software (SoftMax Pro, Molecular Devices) using a 4-parameter logistic fitting algorithm to the standard curve, allowing the determination of antibody concentration in the samples, expressed as EU/ml. More detailed information is provided by Leroux-Roels et al. (22).

### 2.3 Cross-reactive IgG antibodies against non-vaccine VLPs

Plant-produced VLPs of GII.4 OC08154 (2008), GII.4 Washington (2018), and GII.6 Maryland (2018) (Icon Genetics GmbH) were coated onto separate 96-polystyrene plates (Nunc MaxiSorp) by adding 100 µl per well of a coating solution (4 µg/ml). Measurement was performed as described for IgG ELISA in (22). Data analyses were executed without the availability of standards or positive controls. A blank control (Ig-depleted serum) was included on each plate to monitor any unspecific reactions. The readout of OD values was analyzed using a 4-parameter logistic regression model. For further data analysis, the reciprocal of the dilution factor, or c value (inflection point), was used and expressed as the Arbitrary Response Unit (ARU).

The evolutionary history of VP1 protruding domains of norovirus VLPs used in ELISA assays was inferred using MEGA with the Neighbor-Joining method on an alignment generated with MUSCLE (25).

### 2.4 Cell-mediated immune response by intracellular cytokine staining

PBMC were thawed and suspended in RPMI 1640 medium supplemented with Minimum Essential Medium non-essential amino acids: L-glutamine, penicillin/streptomycin, sodium pyruvate, 2-mercapto-ethanol (all from Invitrogen), and fetal bovine serum (Seradigm). Then the PBMCs were incubated *in vitro* with the relevant vaccine antigens or left unstimulated (background condition) in the presence of costimulatory antibodies to CD28 and CD49d. After 2 h, Brefeldin A, a protein transport inhibitor, was added for the subsequent overnight culture. This step ensured the inhibition of cytokine secretion and its accumulation in the expressing cells. On the next day, the cells were stained using fluorochrome-conjugated antibodies to phenotypic markers (CD3, CD4, and CD8), activation (CD40L), cytokine (interferon γ [IFN-γ], interleukin 2 [IL-2], and tumor necrosis factor α [TNF-α]) markers. The samples were analyzed by flow cytometry (BD LSR Fortessa X-20, FlowJo v9.9.6). The vaccine antigens were plant-produced VLPs of GI.4 Chiba 407 (1987) and GII.4 Aomori 2 (2006) provided by Icon Genetics. Both VLP materials were first treated by boiling (at 95°C for 45 min) and then used at a final concentration of 30 µg/ml.

The norovirus-specific CD4^+^ and CD8^+^ T cells were determined by flow cytometry as the CD3^+^CD4^+^ and CD3^+^CD8^+^ events expressing one marker or a combination of markers among CD40L, IFN-γ, IL-2, and TNF-α after *in vitro* stimulation with the vaccine antigen, from which the corresponding signal of the same sample obtained after *in vitro* stimulation with only medium (background) was subtracted. The intracellular cytokine staining (ICS) results were reported as the frequencies (%) of norovirus VLP-specific CD4^+^ or CD8^+^ T cells per parent population. Results below 0.0001% after background subtraction were set at 0.0001%. All analyses have been done with ICS data multiplied by 10,000, resulting in the frequency of cells per million parent cells.

### 2.5 Statistical analysis

Statistical analysis was performed using a 2-way ANOVA or a mixed-effects model with Geisser–Greenhouse correction. Non-parametric tests (Tukey) were used for the correction of multiple comparisons. Correlation analysis was performed using the Spearman test. Statistical analyses and graphs were generated using GraphPad Prism version 9 (GraphPad Software, San Diego, USA).

## 3 Results

### 3.1 Total serum immunoglobulins against vaccine GI.4 and GII.4 VLPs

**Figure 1:**
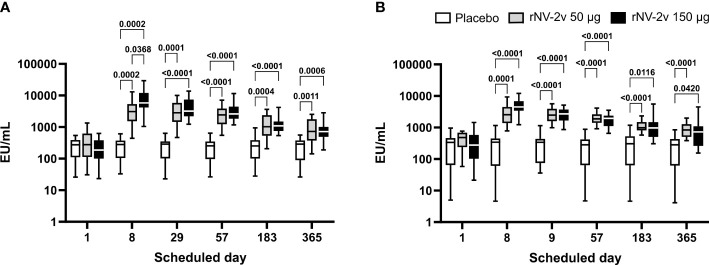
Anti-GI.4 and anti-GII.4 VLP total Ig responses. Total anti-GI.4 and anti-GII.4 VLP Ig responses by scheduled time for each treatment. Vaccine or placebo were administered on days 1 and 29. OD values were processed using a 4-parameter logistic fitting algorithm to the standard curve, allowing the determination of antibody concentration in the samples, expressed as EU/ml. Anti-GI.4 **(A)** and anti-GII.4 **(B)** VLP IgG responses for placebo (white), rNV-2v 50 µg (gray), and 150 µg (black) are shown as boxplots. The horizontal line represents the median; the bottom and top of the box represent the 25^th^ and 75^th^ percentiles of the values, respectively; and the upper and lower error bars represent the maximum and minimum values, respectively. For comparative statistics, all p-values below 0.05 are provided above the boxplots. Ig, immunoglobulin; VLP, virus-like particle.

At both dose levels (50 µg and 150 µg VLPs, respectively), the rNV-2v vaccine candidate elicited a significant increase in total immunoglobulins against GI.4 Chiba 407 (1987) and GII.4 Aomori 2 (2006) VLPs (**Figure 1** and **Table 1**). All subjects displayed detectable anti-GI.4 and anti-GII.4 total serum immunoglobulin titers at baseline (day 1), and no differences in baseline levels were detected between the three groups (rNV-2v 50 µg GI.4 VLPs + 50 µg GII.4 VLPs = rNV-2v 50 µg; rNV-2v 150 µg GI.4 VLPs + 150 µg GII.4 VLPs = rNV-2v 150 µg; placebo). The anti-GI.4 and anti-GII.4 total Ig titers increased after the first vaccination but did not rise further after the second vaccination in both rNV-2v groups. The geometric mean titers of anti-GI.4 and anti-GII.4 VLP remained similar across visits in the placebo group (**Table 1**). Levels are highest at day 8 and decrease over time but remain significantly elevated at day 365 in both dose groups against both antigens (GI.4 and GII.4 VLPs) compared to the placebo group or pre-vaccination levels. A trend of higher levels of total Ig response was observed in the higher dose group (rNV-2v 150 µg) compared to the lower dose group (rNV-2v 50 µg) only at early post-vaccination timepoints.

**Table 1:** Antibody response to vaccine antigens expressed as Geometric Mean Titer (EU/ml) of anti-GI.4 and anti-GII.4 VLP total Ig at scheduled time points for each study group.

			Time point					
Total Ig	Study group	Parameter 95% CI	Day 1	Day 8	Day 29	Day 57	Day 183	Day 365
Anti-GI.4	rNV-2v 50 µg	Geomean*	261.1	2,880.8	2,672.3	2,272.3	1,059.9	737.7
		95% CI	160.6–424.3	1,925.9–4,309.2	1,804.1–3,958.3	1,616.9–3,193.6	718.3–1,563.8	491.1–1,108.0
	rNV-2v 150 µg	Geomean	183.2	6,496.6	3,891.5	2841.6	1,120.5	705.7
		95% CI	126.0–266.4	4,401.5–9,588.8	2,806.9–5,395.3	2,137.7–3,777.3	889.3–1,411.7	534.4–931.9
	Placebo	Geomean	201.6	208.6	200.3	193.2	199.6	183.1
		95% CI	300.7 135.2	305.5 142.5	304.4 131.8	291.7 128.0	305.2 130.6	284.1 118.1
Anti-GII.4	rNV-2v 50 µg	Geomean	381.0	2,492.9	2,409.9	1,936.6	1,079.8	837.0
		95% CI	272.9–532.1	1,785.5–3,480.6	1,902.4–3,052.7	1,590.9–2,357.4	895.6–1,302.0	659.9–1,061.6
	rNV-2v 150 µg	Geomean	241.0	4,429.3	2,490.1	1,701.5	917.9	605.6
		95% CI	137.9–421.1	3,360.0–5,838.7	1,942.2–3,192.5	1,347.6–2,148.3	650.6–1,294.9	402.2–911.9
	Placebo	Geomean	185.1	190.1	234.2	176.6	197.6	168.1
		95% CI	98.6–347.4	98.5–366.7	140.9–389.2	95.0–328.3	104.1–375.1	87.4–323.5

CI, confidence interval; Geomean, geometric mean antibody concentration.

*Geometric mean values are expressed as EU/ml (ELISA units per milliliter).

### 3.2 Serum IgA antibodies against vaccine GI.4 and GII.4 VLPs

**Figure 2:**
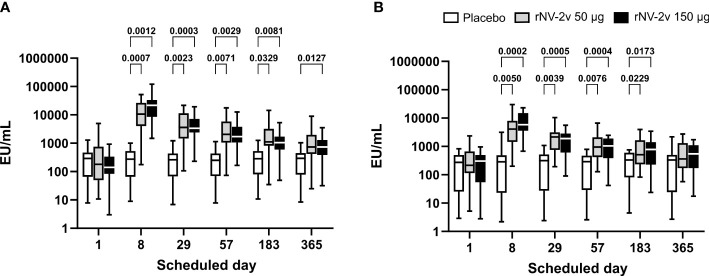
Anti-GI.4 and anti-GII.4 VLP serum IgA responses. Anti-GI.4 and anti-GII.4 VLP IgA responses by scheduled time for each treatment. Vaccine or placebo were administered on days 1 and 29. OD values were processed using a 4-parameter logistic fitting algorithm to the standard curve, allowing the determination of antibody concentration in the samples, expressed as EU/ml. Anti-GI.4 **(A)** and anti-GII.4 **(B)** VLP IgA responses for placebo (white), rNV-2v 50 µg (gray), and 150 µg (black) are shown as boxplots. The horizontal line represents the median; the bottom and top of the box represent the 25^th^ and 75^th^ percentiles of the values, respectively; and the upper and lower error bars represent the maximum and minimum values, respectively. For comparative statistics, all p-values below 0.05 are provided above the boxplots. Ig, immunoglobulin; VLP, virus-like particle.

The magnitude and kinetics of the IgA response against the vaccine antigens, GI.4 Chiba 407 (1987) and GII.4 Aomori 2 (2006) VLPs, are shown in **Table 2** and **Figure 2**. All participants except one displayed detectable anti-GI.4 and anti-GII.4 IgA levels at baseline (day 1), and no relevant differences at baseline were detected between the three groups (rNV-2v 50 µg, rNV-2v 150 µg, placebo). The anti-GI.4 and anti-GII.4 IgA titers increased after the first vaccination but did not rise further after the second vaccination in both rNV-2v groups (**Figure 2**; **Table 2**). The geometric mean titers of anti-GI.4 and anti-GII.4 VLP IgA remained similar across visits in the placebo group (**Table 2**). A trend of higher levels of IgA response was observed in the higher dose group (rNV-2v 150 µg) compared to the lower dose group (rNV-2v 50 µg) only at early post-vaccination timepoints (day 8), but the differences were not statistically significant at any timepoint (p >0.05). Antibody responses against both VLPs, expressed as geometric mean titers, reached peak values on day 8 after the first vaccine dose and then decreased. Antibody concentrations against both VLPs on day 183 still exceeded those measured at baseline (day 1) and placebo control at a statistically significant level (p <0.05). At day 365, only the rNV-2v 150 µg group was still significantly elevated against GI.4 but not GII.4 compared to the placebo group (**Figure 2**).

**Table 2:** Antibody response to vaccine antigens expressed as Geometric Mean Titer (EU/ml) of anti-GI.4 and anti-GII.4 VLP IgA at scheduled time points for each study group.

			Time point					
IgA	Study group	Parameter 95% CI	Day 1	Day 8	Day 29	Day 57	Day 183	Day 365
Anti-GI.4	rNV-2v 50 µg	Geomean*	175.6	7,913.3	3,076.1	1,968.6	1,184.3	765.1
		95% CI	77.6–397.2	3,802.8–1,6467.0	1,535.2–6,163.5	1,000.3–3,874.4	574.2–2,442.6	377.3–1,551.4
	rNV-2v 150 µg	Geomean	139.0	17,767.9	3,586.5	1,879.6	961.6	665.0
		95% CI	77.9–247.8	10,800.8–29,229.3	2,260.6–5,689.9	1,183.7–2,984.7	596.8–1,549.2	410.6–1,076.9
	Placebo	Geomean	159.3	152.2	146.2	152.1	193.6	158.7
		95% CI	76.5–331.7	74.1–312.8	304.3 70.2	74.8–309.4	97.7–383.7	75.9–331.8
Anti-GII.4	rNV-2v 50 µg	Geomean	209.6	3,437.5	1,522.2	966.8	623.9	456.6
		95% CI	105.5–416.2	1,996.2–5,919.6	933.5–2,482.4	587.9–1,589.7	372.1–1,046.1	268.4–776.6
	rNV-2v 150 µg	Geomean	140.5	5,743.1	1,205.4	695.4	489.8	362.0
		95% CI	59.9–329.7	3,552.0–9,285.7	670.7–2,166.4	376.0–1,286.0	250.4–958.0	183.2–715.5
	Placebo	Geomean	131.3	118.6	136.7	131.8	172.9	113.7
		95% CI	54.0–319.1	44.4–317.1	55.2–338.5	55.4–313.4	79.5–376.1	41.7–310.4

CI, confidence interval; Geomean, geometric mean antibody concentration.

*Geometric mean values are expressed as EU/ml (ELISA units per milliliter).

### 3.3 Cross-reactive serum IgG antibodies against non-vaccine VLP elicited by rNV-2v

**Figure 3:**
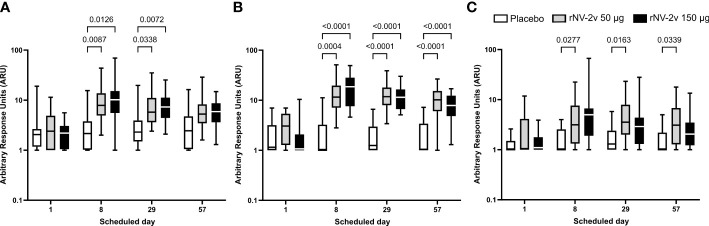
Cross-reactive anti-GII.2, anti-GII.4, and anti-GII.6 VLP IgG responses. Cross-reactive IgG responses against the non-vaccine strain VLP at the scheduled time for each treatment. Vaccine or placebo were administered on days 1 and 29. The cross-reactive IgG arbitrary response unit (ARU) is defined as the reciprocal of the dilution factor or c value (inflection point) of the 4-parameter logistic regression equation. Anti-GII.2 OC08154 (2008) **(A)**, anti-GII.4 Washington (2018) **(B)**, and anti-GII.6 Maryland (2018) **(C)** VLP IgG responses for placebo (white), rNV-2v 50 µg (gray), and rNV-2v 150 µg (black) are shown as boxplots. The horizontal line represents the median; the bottom and top of the box represent the 25^th^ and 75^th^ percentiles of the values, respectively; and the upper and lower error bars represent the maximum and minimum values, respectively. For comparative statistics, all p-values below 0.05 are provided above the boxplots. Ig, immunoglobulin; VLP, virus-like particle.

**Figure 4:**
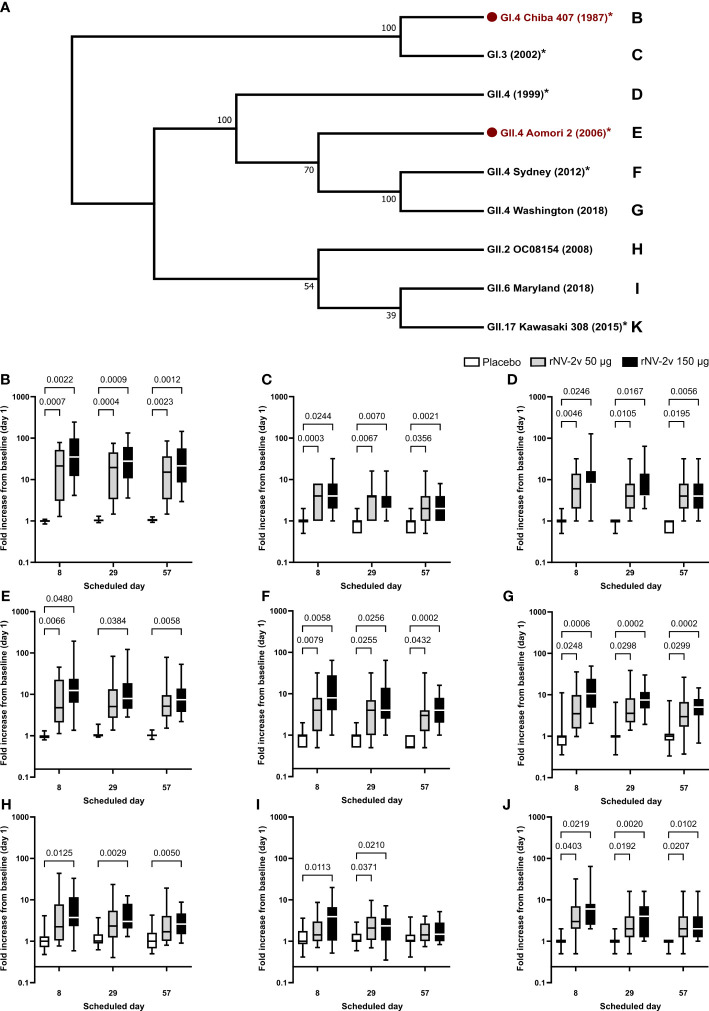
Evolutionary relationships of VP1 protruding domains of norovirus VLPs used in ELISA assays to assess cross-reactive IgG and respective fold-change graphs. In **(A)**, the evolutionary history of VP1 protruding domains of norovirus strains used in ELISA assays to assess IgG response to vaccine and non-vaccine norovirus strains was inferred in MEGA (25). Here, the Neighbor-Joining method on an alignment generated with MUSCLE was used. The bootstrap consensus tree inferred from 10,000 replicates is taken to represent the evolutionary history of the taxa analyzed. Branches corresponding to partitions reproduced in less than 50% of bootstrap replicates were collapsed. The percentage of replicate trees in which the associated taxa clustered together in the bootstrap test (10,000 replicates) is shown next to the branches. The evolutionary distances were computed using the Poisson correction method and are in units of the number of amino acid substitutions per site. The analysis involved nine amino acid sequences. All positions containing gaps and missing data were eliminated. There were a total of 290 positions in the final dataset. Norovirus genogroup (GI or GII), genotype, strain designation, and year of isolation are provided. Vaccine strains are indicated in red with a red dot. * = analyses with detailed data published in Leroux-Roels et al. (22). The letters next to the strain designation refer to the panel providing the fold increase of the IgG from baseline (day 1) on the scheduled day for the respective norovirus strain. Fold increase from baseline (day 1) calculated from ELISA results is provided for norovirus strains GI.4 Chiba 407 (1987) **(B)**, GI.3 (1999) **(C)**, GII.4 (1999) **(D)**, GII.4 Aomori 2 (2006) **(E)**, GII.4 Sydney (2012) **(F)**, GII.4 Washington (2018) **(G)**, GII.2 OC08154 (2008) **(H)**, GII.6 Maryland (2018) **(I)**, and GII.17 Kawasaki 308 (2015) **(J)**. For comparative statistics, all p-values below 0.05 are provided above the boxplots. VP1, norovirus major capsid protein; VPL, virus-like particle; ELISA, enzyme-linked immunosorbent assay; Ig, immunoglobulin.

The rNV-2v vaccine elicited cross-reactive IgG antibodies against non-vaccine VLPs. The cross-reactive IgG titers of anti-GI.3 (2002), anti-GII.4 (1999), anti-GII.4 Sydney (2012), and anti-GII.17 Kawasaki 308 (2015) have already been reported by Leroux-Roels et al. (22), while the current report expands this by examining responses to GII.2 OC08154 (2008), GII.4 Washington (2018), and GII.6 Maryland (2018) VLPs. These results are provided in **Figures 3**, **4**, and **Tables 3**, **4**.

**Table 3:** Antibody response to non-vaccine antigens expressed as Geometric Mean of arbitrary response units (ARU) of anti-GII.2, anti-GII.4, and anti-GII.6 VLP IgG at scheduled time points for each study group.

			Time point			
IgG	Study group	Parameter 95% CI	Day 1	Day 8	Day 29	Day 57
Anti-GII.2 OC08154 (2008)	rNV-2v 50 µg	Geomean*	2.54	7.90	6.66	5.46
		95% CI	1.72–3.75	5.52–11.31	4.83–9.19	3.95–7.54
	rNV-2v 150 µg	Geomean	2.01	9.51	7.05	5.24
		95% CI	1.54–2.61	6.21–14.58	5.27–9.43	3.94–6.96
	Placebo	Geomean	2.18	2.22	2.54	2.45
		95% CI	1.55–3.06	1.57–3.14	1.79–3.61	1.68–3.59
Anti-GII.4 Washington (2018)	rNV-2v 50 µg	Geomean	2.75	11.40	11.54	8.83
		95% CI	2.01–3.77	8.07–16.11	8.72–15.27	6.17–12.63
	rNV-2v 150 µg	Geomean	1.56	16.55	11.18	6.92
		95% CI	1.16–2.11	11.70–23.41	8.83–14.17	4.98–9.62
	Placebo	Geomean	1.71	1.71	1.74	1.77
		95% CI	1.26–2.33	1.22–2.40	1.27–2.40	1.25–2.51
Anti-GII.6 Maryland (2018)	rNV-2v 50 µg	Geomean	1.88	3.35	3.91	3.11
		95% CI	1.27–2.76	2.16–5.19	2.62–5.82	2.06–4.71
	rNV-2v 150 µg	Geomean	1.39	4.47	2.84	2.28
		95% CI	1.12–1.72	2.74–7.31	1.91–4.23	1.65–3.15
	Placebo	Geomean	1.27	1.48	1.55	1.48
		95% CI	1.08–1.50	1.15–1.90	1.21–1.97	1.14–1.91

CI, confidence interval; Geomean, geometric mean antibody concentration.

*Geometric mean values are expressed as arbitrary response units (ARU) deﬁned as the reciprocal of the dilution factor or c value (inflection point).

**Table 4:** Antibody response to vaccine and non-vaccine antigens expressed as median of fold increase from baseline (day 1) for anti-VLP IgG at scheduled time points for each study group.

			Time point		
IgG Fold Increase	Study group	Median	Day 8	Day 29	Day 57
		Min - Max			
Anti-GI.4 Chiba 407 (1987)	rNV-2v 50 µg	Median	**21.3**	**19.5**	**15.1**
		Min - Max	1.3 - 78.3	1.5 - 75.0	1.5 - 85.4
	rNV-2v 150 µg	Median	**35.2**	**27.7**	**21.4**
		Min - Max	4.1 - 245.3	3.6 - 133.5	2.9 - 145.6
	Placebo	Median	**1.0**	**1.0**	**1.0**
		Min - Max	0.8 – 1.1	0.9 – 1.3	0.9 – 1.3
Anti-GI.3 (2002)	rNV-2v 50 µg	Median	**4.0**	**4.0**	**2.0**
		Min - Max	1.0 – 8.0	1.0 – 16.0	0.5 – 16.0
	rNV-2v 150 µg	Median	**4.0**	**2.0**	**2.0**
		Min - Max	1.0 – 32.0	1.0 – 32.0	1.0 – 8.0
	Placebo	Median	**1.0**	**1.0**	**1.0**
		Min - Max	0.5 – 2.0	0.5 – 2.0	0.5 – 2.0
Anti-GII.4 (1999)	rNV-2v 50 µg	Median	**6.0**	**4.0**	**4.0**
		Min - Max	1.0 – 32.0	1.0 – 32.0	1.0 – 32.0
	rNV-2v 150 µg	Median	**8.0**	**4.0**	**4.0**
		Min - Max	1.0 – 128.0	2.0 – 64.0	1.0 – 32.0
	Placebo	Median	**1.0**	**1.0**	**1.0**
		Min - Max	0.5 – 2.0	0.5 – 1.0	0.5 – 1.0
Anti-GII.4 Aomori 2 (2006)	rNV-2v 50 µg	Median	**4.8**	**5.1**	**5.2**
		Min - Max	1.1 - 45.3	1.4 - 83.0	1.5 - 78.8
	rNV-2v 150 µg	Median	**12.4**	**8.0**	**7.4**
		Min - Max	1.4 - 193.0	2.8 - 121.3	2.2 - 53.3
	Placebo	Median	**0.9**	**1.0**	**1.0**
		Min - Max	0.8 – 1.3	0.9 – 1.9	0.8 – 1.4
Anti-GII.4 Sydney (2012)	rNV-2v 50 µg	Median	**4.0**	**4.0**	**3.0**
		Min - Max	0.5 - 32	0.5 - 32	0.5 - 32
	rNV-2v 150 µg	Median	**8.0**	**4.0**	**4.0**
		Min - Max	1.0 – 64.0	1.0 – 64.0	1.0 – 16.0
	Placebo	Median	**1.0**	**1.0**	**0.5**
		Min - Max	0.5 – 2.0	0.5 – 2.0	0.5 – 1.0
Anti-GII.4 Washington (2018)	rNV-2v 50 µg	Median	**3.5**	**3.6**	**3.0**
		Min - Max	1.0 - 35.6	1.4 - 38.8	0.4 - 26.5
	rNV-2v 150 µg	Median	**10.7**	**7.5**	**5.1**
		Min - Max	2.0 - 49.3	1.9 - 30.1	0.7 - 14.7
	Placebo	Median	**1.0**	**1.0**	**1.0**
		Min - Max	0.4 - 11.2	0.4 - 6.6	0.3 - 7.2
Anti-GII.2 OC08154 (2008)	rNV-2v 50 µg	Median	**2.2**	**2.3**	**1.7**
		Min - Max	0.8 - 43.7	0.4 - 23.4	0.8 - 19.2
	rNV-2v 150 µg	Median	**3.7**	**3.0**	**2.6**
		Min - Max	0.6 - 33.0	1.3 - 12.5	0.9 - 8.8
	Placebo	Median	**1.0**	**1.0**	**1.0**
		Min - Max	0.5 - 4.1	0.6 - 3.7	0.5 - 4.3
Anti-GII.6 Maryland (2018)	rNV-2v 50 µg	Median	**1.4**	**2.1**	**1.4**
		Min - Max	0.7 - 8.7	0.7 - 9.7	0.7 - 4.0
	rNV-2v 150 µg	Median	**3.9**	**2.4**	**1.5**
		Min - Max	0.5 - 19.9	0.4 - 7.2	0.8 - 5.2
	Placebo	Median	**1.0**	**1.0**	**1.0**
		Min - Max	0.4 - 3.6	0.6 - 2.9	0.4 - 3.8
Anti-GII.17 Kawasaki (2015)	rNV-2v 50 µg	Median	**3.0**	**2.0**	**2.0**
		Min - Max	0.5 – 32.0	0.5 – 16.0	0.5 – 16.0
	rNV-2v 150 µg	Median	**6.0**	**4.0**	**2.0**
		Min - Max	2.0 – 64.0	1.0 – 16.0	1.0 – 16.0
	Placebo	Median	**1.0**	**1.0**	**1.0**
		Min - Max	0.5 – 2.0	0.5 – 2.0	0.5 – 1.0

For GII.2 OC08154 (2008) and GII.4 Washington (2018), the IgG titers were evaluated at pre-vaccination (day 1), day 8, day 29 (before the second vaccination), and on day 57. Cross-reactive IgG titers increased after the first vaccination but did not rise further after the second vaccination in both rNV-2v groups (**Figure 3** and **Table 3**). The geometric mean titers of anti-GII.2 and anti-GII.4 VLP IgG remained similar across visits in the placebo group (**Table 3**). A trend of higher levels of IgG response was observed in the higher dose group (rNV-2v 150 µg) compared to the lower dose group (rNV-2v 50 µg) only at early post-vaccination timepoints (day 8), but the differences were not statistically significant at any timepoint (p >0.05). Antibody responses against GII.2 OC08154 (2008) and GII.4 Washington (2018) VLPs expressed as cross-reactive IgG arbitrary response units (ARU) and fold-increase reached peak values on day 8 after the first vaccine dose and declined thereafter. Antibody response against GII.4 Washington (2018) VLPs was higher and remained significantly elevated compared to baseline and placebo until day 57. Response against GII.2 OC08154 (2008) VLPs seemed weaker and was only significant until day 29 (p <0.05). The cross-reactive IgG response against GII.6 Maryland (2018) was weaker than that for the other two VLPs analyzed here. Significant differences from baseline were only detected for the lower dose group (rNV-2v 50 µg) at all three post-vaccination time points considered (**Figure 3**).

The evolutionary relationships of the VP1 protruding domains of norovirus VLPs used in ELISA assays to assess cross-reactive IgG are illustrated in **Figure 4**. As data from different ELISA assays using different antigens cannot be evaluated quantitatively, a qualitative comparison of the cross-reactive IgG data calculating fold increases (FI) from baseline at day 1 was performed for timepoints day 8, day 29, and day 57 (**Figure 4** and **Table 4**). The rNV-2v vaccine elicited cross-reactive IgG antibodies against non-vaccine VLPs, which were more pronounced for strains of the same genotype (closely related VP1 sequences) as the GII.4 vaccine strain, i.e., GII.4 (1999), GII.4 Sydney (2012), and GII.4 Washington (2018), and less for non-vaccine genotypes (i.e., GI.3 [2002], GII.2 OC08154 [2008], GII.6 Maryland [2018], and GII.17 Kawasaki 308 [2015]). The lowest response was observed for GII.6 Maryland (2018) (**Figures 3**, **4** and **Tables 3**, **4**). At most post-vaccination timepoints, the differences in anti-GI.3 (2002), anti-GII.2 OC08154 (2008), anti-GII.4 (1999), anti-GII.4 Sydney (2012), anti-GII.4 Washington (2018), and anti-GII.17 Kawasaki 308 (2015) IgG titers between the vaccine and placebo groups, respectively, were statistically significant (p <0.05) [(22), **Figures 3**, **4**]. The differences between the two rNV-2v groups, i.e., 50 µg vs. 150 µg were not statistically significant (p >0.05) at any post-vaccination timepoint [**Figures 3**, **4** and (22)]. The anti-GI.3 (2002), anti-GII.2 OC08154 (2008), anti-GII.4 (1999), anti-GII.4 Sydney (2012), anti-GII.4 Washington (2018), anti-GII.6 Maryland (2018), and anti-GII.17 Kawasaki 308 (2015) IgG titers increased after the first vaccination but did not increase further after the second vaccination in both rNV-2v groups.

### 3.4 GI.4- and GII.4-specific cellular immune responses as measured by intracellular cytokine staining

**Figure 5:**
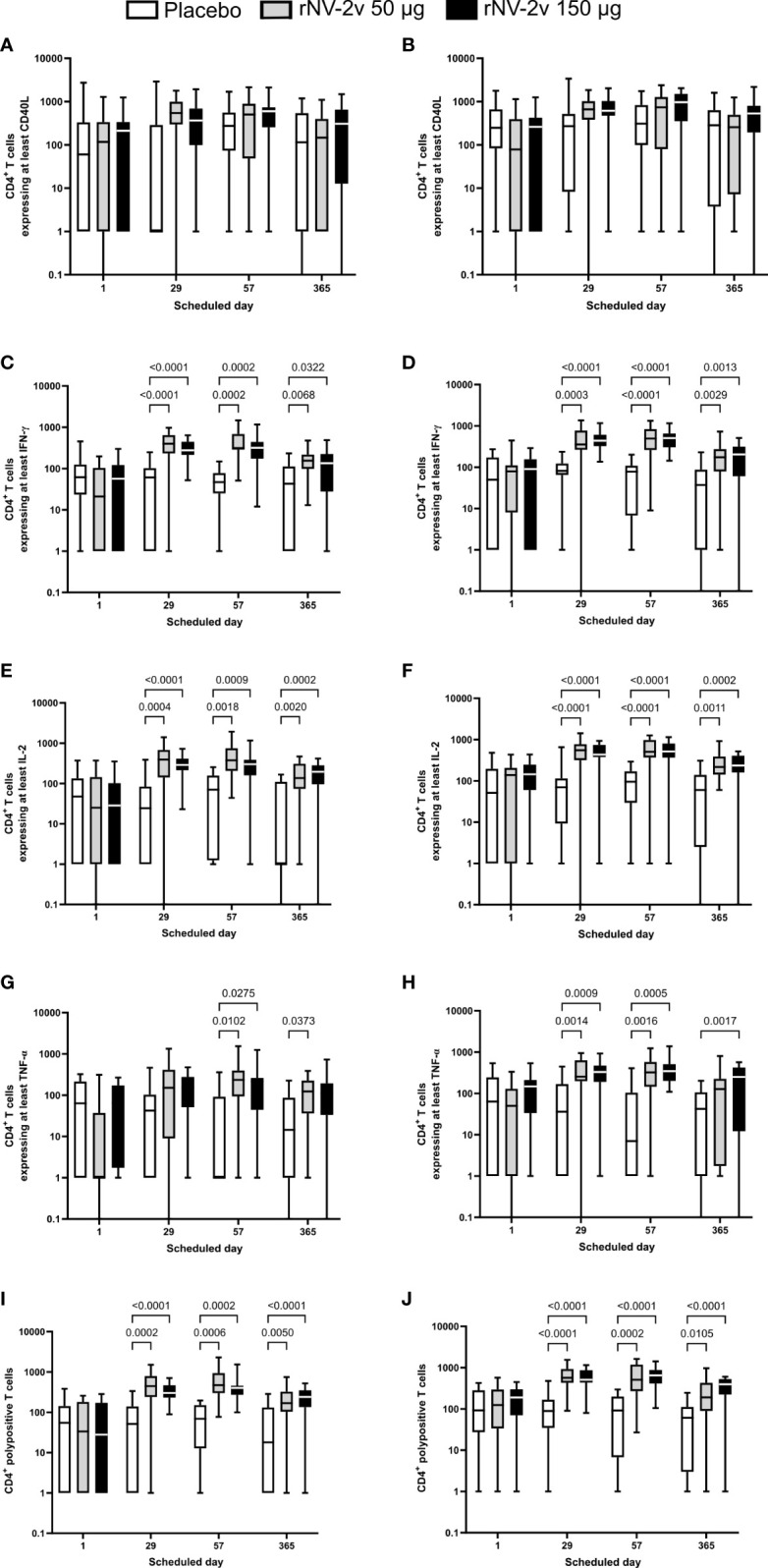
Intracellular cytokine staining—CD4^+^ T cells expressing at least one or a combination of markers after stimulation with GI.4 and GII.4 VLPs. ICS data shown here are the frequencies of norovirus VLP-specific CD4^+^ T cells per million parent cells. Boxplots are provided for CD4^+^ T cells expressing at least CD40L after stimulation with GI.4 Chiba 407 (1987) **(A)** or GII.4 Aomori 2 (2006) **(B)** VLPs, for CD4^+^ T cells expressing at least interferon γ (IFN-γ) after stimulation with GI.4 **(C)** or GII.4 **(D)** VLPs, for CD4^+^ T cells expressing at interleukin 2 (IL-2) after stimulation with GI.4 **(E)** or GII.4 **(F)** VLPs, and for CD4^+^ T cells expressing at least tumor necrosis factor α (TNF-α) after stimulation with GI.4 **(G)** or GII.4 **(H)** VLPs. CD4^+^ polypositive T cells are defined as those that express at least two of the following immune markers: CD40L, IFN-γ, IL-2, and TNF-α. CD4^+^ polypositive T cells after stimulation with GI.4 **(I)** and GII.4 **(J)**. Boxplots are shown for placebo (white), rNV-2v 50 µg (gray), and 150 µg (black) for the respective scheduled times. The horizontal line in the boxplots represents the median; the bottom and top of the box represent the 25^th^ and 75^th^ percentiles of the values, respectively; and the upper and lower error bars represent the maximum and minimum values, respectively. For comparative statistics, all p-values below 0.05 are provided above the boxplots. VLP, virus-like particle.

Most subjects displayed vaccine strain-specific (GI.4 Chiba 407 [1987] and GII.4 Aomori 2 [2006]) cell-mediated responses at baseline (day 1), detected before by lymphoproliferation (22) and now investigated in more detail by intracellular cytokine staining (ICS) (**Table 5** and **Figure 5**). The norovirus-specific CD4^+^ and CD8^+^ T cells were determined by flow cytometry as CD3^+^CD4^+^ and CD3^+^CD8^+^ cells expressing at least one of the following markers: CD40L, IFN-γ, IL-2, and TNF-α.

**Table 5:** CD4^+^ polypositive T cells after stimulation with GI.4 and GII.4 VLPs expressed as Geometric Mean of the cell count at scheduled time points for each study group.

			Time point			
CD4^+^ polypositive	Study group	Parameter 95% CI	Day 1	Day 29	Day 57	Day 365
GI.4 Chiba 407 (1987)	rNV-2v 50 µg	Geomean*	22.2	337.9	457.6	114.9
		95% CI	7.5 - 65.8	158.6 - 719.9	305.7 - 685.0	50.8 - 260.3
	rNV-2v 150 µg	Geomean	13.8	314.4	378.6	170.6
		95% CI	4.4 - 43.3	247.8 - 399.1	286.5 - 500.3	87.9 - 331.0
	Placebo	Geomean	17.6	21.9	32.6	10.2
		95% CI	5.6 - 55.9	7.8 - 61.8	13.1 - 81.1	3.3 - 32.0
GII.4 Aomori 2 (2006)	rNV-2v 50 µg	Geomean	56.6	585.3	459	133.2
		95% CI	20.0 - 160.8	428.4 - 799.8	273.9 - 769.3	60.4 - 293.9
	rNV-2v 150 µg	Geomean	96.8	515.5	581.3	210.8
		95% CI	42.7 - 219.2	379.3 - 700.5	437.4 - 772.5	96.9 - 458.5
	Placebo	Geomean	50	42.8	36.9	27.7
		95% CI	18.2 - 137.2	16.5 - 110.8	13.0 - 105.0	10.3 - 74.6

CI, confidence interval; Geomean, geometric mean of the cell count.

*Geometric mean values are expressed as cell count (frequencies per million parent cells.

CD8^+^ T cell responses were below the lower limit of quantification set at 0.0335% CD8^+^ polypositive cells (26) and therefore interpreted as very low to negative. This was not surprising as the PBMC had been stimulated overnight with boiled VLPs and, e.g., not with peptide libraries representing the VP1 proteins of GI.4 Chiba 407 (1987) and GII.4 Aomori 2 (2006). Therefore, the analysis was focused on CD4^+^ T cells positive for at least one marker and CD4^+^ polypositive T cells (**Figure 5** and **Table 5**).

CD40L (or CD154) is a co-stimulatory molecule expressed on almost all activated CD4^+^ T cells and is therefore considered a CD4^+^ T cell activation marker (27). An increased number of CD40L-expressing CD4^+^ T cells was observed after the first vaccination (Day 29) in all groups for both VLPs but was not statistically significant (p >0.05) (**Figure 5**).

CD4^+^ T cells expressing the cytokine IFN-γ are known to be key anti-viral effectors and a distinctive feature of a Th1 response (28). A significant increase in the number of IFN-γ expressing CD4^+^ T cells was observed after the first vaccination (day 29), with a similar to slightly increased response after the second vaccination (day 57) for both GI.4 and GII.4 VLPs. Responses wane by day 365 but are still significantly higher than the observed baseline responses (day 1) and placebo. Similar patterns were observed for the low (rNV-2v 50 µg) and high-dose (rNV-2v 150 µg) groups, with a trend of less dispersed responses in the high-dose group (**Figure 5**).

IL-2 is a key cytokine for T cell development, survival, and function (29). An increased number of IL-2-producing CD4^+^ T cells was observed after the first vaccination (day 29), with a similar to slightly increased response after the second vaccination (day 57) for both GI.4 and GII.4 VLPs. Responses wane at day 365 but are still higher than the observed baseline responses (day 1) or placebo. Similar patterns are observed for the low (rNV-2v 50 µg) and high (rNV-2v 150 µg) dose groups (**Figure 5**).

TNF-α is a pro-inflammatory cytokine, characterized by a broad spectrum of functions that also include cytotoxic and anti-viral effects (30). An increase in TNF-α expressing CD4^+^ T cells was observed after the first vaccination (day 29), but it was less pronounced for GI.4 compared to GII.4. A similar to slightly higher response was observed after the second vaccination (day 57) for both GI.4 and GII.4 VLPs. At day 365, responses waned, especially in the GII.4 low-dose group, where baseline levels were nearly reached (**Figure 5**).

In both the low (rNV-2v 50 µg) and high (rNV-2v 150 µg) dose groups, a statistically significant increase in polypositive CD4^+^ T cell responses was observed after the first vaccination (day 29) that persisted up to day 57. Responses waned slightly, as measured on day 365. No booster effect was observed after the second dose (**Figure 5** and **Table 5**).

### 3.5 Correlation analysis of humoral and cell-mediated immune responses elicited by rNV-2v administration

A correlation analysis was performed using the Spearman test on all available data from the low (rNV-2v 50 µg) and high (rNV-2v 150 µg) dose groups for all assays done (the placebo group was not considered here).

**Figure 6:**
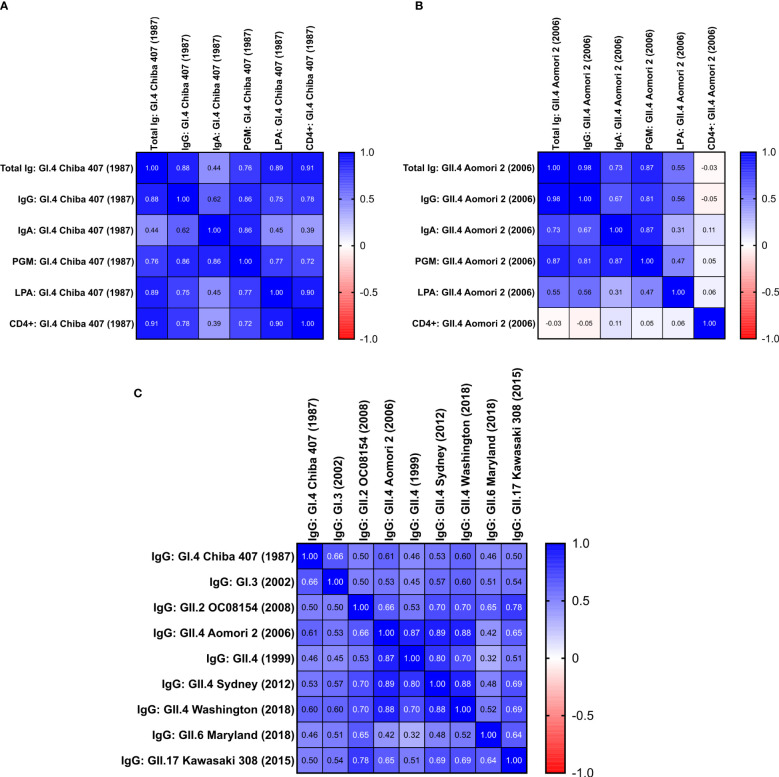
Correlation between the different humoral and cell-mediated immune responses triggered by rNV-2v. Correlation matrices are provided as heat maps for responses against vaccine strains GI.4 Chiba 407 (1987) **(A)** and GII.4 Aomori 2 (2006) **(B)** and IgG responses against vaccine and non-vaccine VLPs **(C)**. The placebo group was not considered for the analysis. Values are the Spearman correlation coefficients of data obtained from low (rNV-2v 50 µg) and high (rNV-2v 150 µg) dose groups. **(A)** Calculated p-values for Spearman correlation coefficients ≥0.45 were below 0.05. **(B)** Calculated p-values for Spearman correlation coefficients ≥0.31 were below 0.05. **(C)** All calculated p-values for Spearman correlation coefficients were below 0.05. PGM, Pic gastric mucin blocking assay, see (22) for more details; CD4+ = CD4^+^ polypositive T cells from intracellular cytokine staining; Ig, immunoglobulin; VLP, virus-like particle.

The analysis revealed a correlation between all IgG titers for the GI and GII vaccines and non-vaccine VLPs. The strongest correlation was detected between reactions against the different VLPs derived from strains of the GII.4 genotype and the weakest between GI and GII VLPs and for GII.6 Maryland (2018) (**Figure 6**).

When analyzing the humoral (total Ig, IgG, IgA, Pig Gastric Mucin [PGM] blocking assay) and cell-mediated responses (lymphoproliferation assay [LPA], CD4^+^ polypositive T cells by ICS) against the vaccine strains (GI.4 Chiba 407 [1987] and GII.4 Aomori 2 [2006]), the highest and very strong correlation was observed between IgG and total Ig titers (**Figure 6**). The lowest correlation between the total Ig, IgG, and IgA responses was found for IgA. On the other hand, the IgA data correlated well with the results obtained in the PGM blocking assay [detailed PGM blocking assay data are available in Leroux-Roels et al., 22)]. Generally, the data obtained from the PGM blocking assay correlated well with other humoral responses (**Figure 6**).

For the vaccine GI.4 VLPs, the data for humoral responses correlated well with cellular immunity parameters, except for IgA (**Figure 6**). While for GI.4, a good correlation between lymphoproliferation after stimulation (LPA) and total Ig and IgG titers is observed, the correlation is significantly lower for the data obtained for the vaccine GII.4 VLPs. This was similar for polypositive CD4^+^ T cells (as determined by ICS), where a good correlation is observed between total Ig and IgG data for GI.4 but no correlation for the GII.4 data (**Figure 6**). Also, no correlation is observed between LPA and ICS for GII.4 (Spearman r = 0.057, p = 0.48), whereas a strong correlation between both cellular parameters is observed for GI.4 (Spearman r = 0.9, p = 0.0000015).

## 4 Discussion

The investigational vaccine rNV-2v elicits strong humoral and cellular immune responses against both antigens present in the vaccine, GI.4 Chiba 407 (1987) and GII.4 Aomori 2 (2006). In addition to the vaccine-induced increase of antigen-specific IgG reported previously (22), significant rises in antigen-specific serum IgA and total Ig are shown here. No significant differences were observed between the low dose (rNV-2v 50 µg) and high dose (rNV-2v 150 µg) groups, except for total Ig against GI.4 at day 8. No booster effect of the second vaccine administration could be observed, as expected in a pre-exposed population and in line with findings for other VLP-based vaccine candidates (19–22). Anti-GI.4 Chiba 407 (1987) and anti-GII.4 Aomori 2 (2006) total Ig and IgA titers reached a peak at day 8 and declined over time but remained significantly elevated at day 365 or day 183, respectively. In general, the IgA response to GII.4 appears to be less strong than the IgA response to GI.4. Also, the persistence of anti-GII.4 is less than that of GI.4. However, the IgA response might be underestimated here, especially against the dominant GII.4 genotype with more frequent exposure in the adult population. A high and specific IgG response detected as a major humoral response to norovirus in a pre-exposed population could interfere with IgA detection, as described in the assessment of responses against other pathogens (31, 32).

Pathogen-specific immunity at the mucosal sites is best elicited by the mucosal application of the antigen. Examples of vaccines that elicit a protective mucosal IgA response are the oral polio vaccine, the oral rotavirus vaccine, and the live-attenuated nasal influenza vaccine (33, 34). Parenteral immunization can also be effective in enhancing mucosal antibody responses, but this might require prior mucosal priming *via* natural infection or vaccination with live, attenuated pathogens. For poliovirus vaccines, it was demonstrated that parenteral IPV vaccination could boost systemic and mucosal IgA responses in previously OPV-vaccinated individuals only (35). Since all participants in this study have been previously exposed to norovirus, a similar mechanism can be invoked to explain the strong increases in antigen-specific IgA levels. These increases in serum IgA may reflect an increase in gastro-intestinal secretory IgA. The mucosal immune response with respect to protection from norovirus gastroenteritis and reduction of viral replication in the gut needs to be evaluated more extensively in future studies by the detection and quantification of antigen-specific IgA and α4β7-expressing, gut-selective B cells in the circulation and by the measurement of secretory IgA in saliva and/or feces (13, 14, 33, 36–39). In addition to its virus neutralizing activity, IgA at the mucosal sites can mediate antibody-dependent cell-mediated cytotoxicity (ADCC) (40, 41) and play an active role in host-pathogen defense by activating myeloid cells through diverse receptors, including its Fc receptor, FcαRI (CD89) (42).

Another assessment of the humoral response that is often debated is the analysis of the blocking of norovirus binding to histo-blood group antigens (HBGA) (13, 14). Norovirus binding to HBGA (and surrogates like pig gastric mucin) might be attributed to its ability to bind biological surfaces to enhance viral spread and infection. The binding of the norovirus surface to HBGA is weak (43) and HBGA might only act as one of several attachment factors (44). As of now, the receptor for norovirus on the human cell surface could not be identified (44). Notably, proteinaceous receptors of animal viruses related to human noroviruses have been identified, e.g., CD300lf for murine norovirus (45). Furthermore, it is now possible to infect human cell models with norovirus, allowing more elaborate screening approaches to identify receptor candidates, e.g., genome-wide CRISPR screens (46, 47). Identifying the receptor for human norovirus will enhance the identification and development of potential correlates of protection. However, as HBGA binding most likely only enhances but may not determine norovirus infection, the biological relevance of such assays for vaccine development is debatable. As has also been observed by others (48), strong correlations between norovirus-specific serum IgA, IgG, and total Ig concentrations and HBGA-blocking antibodies (PGM) are observed (correlation coefficients varying between 0.76 and 0.87 for GI.4 and GII.4).

ICS was used to examine in more detail the significant cell-mediated response to rNV-2v previously demonstrated by LPA (22). ICS was able to detect an antigen-specific CD4^+^ polypositive T cell response to rNV-2v. However, no CD8^+^ response was observed. A CD8^+^ response can be expected in the context of norovirus infection (49, 50), but it was also absent from a similar VLP-based vaccine and analytical setup (20). This may be attributed to the use of VLPs as *in vitro* stimulating antigens rather than a library of overlapping peptides representing VP1 proteins. The CD4^+^ polypositive (or polyfunctional) T-cell response detected here is generally important for an effective viral vaccine and often correlates with better protection against viral diseases (51). Therefore, with respect to correlates of protection, cell-mediated responses should be explored more thoroughly, as recently shown for flu vaccines (52–55). Cell-mediated responses elicited by norovirus vaccine candidates may contribute to vaccine-induced protection and should be investigated more thoroughly, as suggested by our data and other studies (20, 56–58). Interestingly, the correlation between GII.4 cell-mediated and humoral responses was low or lacking, in contrast to GI.4-specific immune responses. Similar results were described in adults (57) and children (56) after norovirus infection. This effect might be attributed to differences in the pre-existing immunity and higher frequency of reinfection with GII.4 strains, differences in interaction with the host immune system between different norovirus genogroups, genotypes, or even strains, or the experimental setup as such, and warrants further examination.

It is important to stress that the level of cross-reactivity of the vaccine-induced IgG antibodies cannot be evaluated quantitatively, as no standardization is possible. To assess the different assays for cross-reactive IgG responses [described here and in (22)], which differ significantly in method and used VLP material, a fold increase over baseline on day 1 was used. In an early clinical stage and especially in a pre-exposed population, fold increases can only provide a rough indication for the level of immune responses and should be interpreted with caution (59). Additionally, the Spearman correlation of the obtained data was used for evaluation. The applied methodology allows for a qualitative assessment of the cross-reactive IgG response elicited by rNV-2v. A significantly elevated cross-reactive IgG response was elicited by rNV-2v for all analyzed VLPs composed of the major capsid protein VP1 of strains GI.3 (2002), GII.2 OC08154 (2008), GII.4 (1999), GII.4 Sydney (2012), GII.4 Washington (2018), GII.6 Maryland (2018), and GII.17 Kawasaki 308 (2015), respectively. Responses were more pronounced for VLPs derived from strains of the same genotype as the vaccine strain GII.4 (GII.4 [1999], GII.4 Sydney [2012], GII.4 Washington [2018]) and lesser for the others (GI.3 [2002], GII.2 OC08154 [2008], GII.6 Maryland [2018]). Additionally, there is a very strong correlation observed intra-genotype for GII.4, with Spearman correlation coefficients of 0.70–0.89. However, the correlations across genotypes (e.g., 0.53–0.70 for GII.4 vs. GII.2 and 0.51–0.60 for GII.4 vs. GII.17) are high as well. The lowest levels of cross-reactive IgG and correlation to other data (0.32–0.64) were observed for GII.6 Maryland (2018). The nature and number of previous infections with norovirus may have an impact on the cross-reactivity profile of antibodies against VP1. However, the limited number of participants in this phase I study, the age-homogeneous character of the cohort, and most importantly, the lack of information on their norovirus infection history precludes any analysis of this kind.

Cross-protection due to cross-reactive responses triggered by norovirus vaccine strains is likely, as discussed for the bivalent GI.1/GII.4 VLP-based vaccine evaluated by Takeda, which showed levels of cross-protection against disease caused by GII.2 strains (18). Also, in this study, we see a significantly elevated level of GII.2 cross-reactive IgG triggered by the GI.4/GII.4 vaccine candidate rNV-2v, which also correlates well with the IgG response to the GII.4 vaccine strain (correlation coefficient 0.66). Nevertheless, multivalent vaccine designs are preferred to cover a wide range of presently circulating and emerging genotypes and strains responsible for infection (13, 60, 61). Furthermore, it is promising that a very reliable immune response is triggered for strains of the same genotype as the vaccine strain, which was shown in this study for the dominant GII.4 genotype and for strains spanning three decades of isolation from patients. On the other hand, the evaluated GII.6 Maryland (2018) showed the weakest cross-reactive IgG levels and a lower correlation to other data. Therefore, GII.6 VLPs may have to be included in future vaccine compositions if protection against this genotype is desired. In conclusion, a multivalent vaccine is expected to broaden the spectrum of reactivities and improve protection against present and future emerging strains.

## 5 Conclusion

A significant increase in humoral (total Ig, IgG, and IgA) and CD4^+^ polypositive T-cell responses was triggered by the VLP-based norovirus candidate rNV-2v, which is formulated without adjuvants. No booster effect was observed after the second dose (administered on day 29) in the pre-exposed adult study population. Furthermore, a cross-reactive immune response was elicited, as shown by IgG titers against GI.3 (2002), GII.2 OC08154 (2008), GII.4 (1999), GII.4 Sydney (2012), GII.4 Washington (2018), GII.6 Maryland (2018), and GII.17 Kawasaki 308 (2015). rNV-2v may therefore also convey protection against norovirus strains not included in the vaccine. Nevertheless, multivalent vaccine designs to broaden the spectrum of reactivities are preferable to cover the multitude of present and emergent strains responsible for norovirus gastroenteritis. Potential correlates of protection might encompass IgA, and cross-reactive humoral responses, and CD4^+^ polypositive T cells. However, further work is required, as no clear correlation between immune response and protection from severe norovirus gastroenteritis has been identified so far. The combination of humoral and cell-mediated correlates needs to be explored more thoroughly in the context of norovirus gastroenteritis and vaccination.

## Data availability statement

The original contributions presented in the study are included in the article. Further inquiries can be directed to the corresponding author.

## Ethics statement

The studies involving human participants were reviewed and approved by the Central Ethics Committee, OLV Ziekenhuis Aalst, Belgium and the local Ethics Committee of the Ghent University Hospital, Belgium. The participants provided their written informed consent to participate in this study.

## Author contributions

IL-R was the Principal Investigator of this study. BJ and GL-R were co-investigators of this study. GW and YJ were responsible for the humoral and cell-mediated immunoassays at the CEVAC laboratory. Concept and design: GW, YJ, FJ, AD, GL-R, VK, IL-R, and FT. Acquisition, analysis, or interpretation of data: GW, YJ, BJ, FJ, AD, GL-R, IL-R, and FT. Administrative, technical, or material support: GW, YJ, BJ, FJ, AD, GL-R, VK, IL-R, and FT. GL-R and FT drafted the manuscript. All authors listed have made a substantial, direct, and intellectual contribution to the work and approved it for publication.
